# The epidemiology of infectious gastroenteritis related reactive arthritis in U.S. military personnel: a case-control study

**DOI:** 10.1186/1471-2334-10-266

**Published:** 2010-09-13

**Authors:** Jennifer A Curry, Mark S Riddle, Robert P Gormley, David R Tribble, Chad K Porter

**Affiliations:** 1 Uniformed Services University of the Health Sciences, Bethesda, Maryland, USA; 2 Naval Medical Research Center, Silver Spring, Maryland, USA

## Abstract

**Background:**

Reactive arthritis (ReA) is a recognized sequela of infectious gastroenteritis (IGE). However, the population-based incidence of IGE-related ReA is poorly defined, and the risk of disease has not previously been characterized in a military population. The intent of this study was to provide estimates of the incidence and morbidity associated with IGE-related ReA in the U.S. military population.

**Methods:**

Using active duty US military medical encounter data from the Defense Medical Surveillance System, we conducted a matched case-control study to assess the risk of ReA following IGE. Both specific and nonspecific case definitions were utilized to address ICD-9 coding limitations; these included specific ReA (Reiter's Disease or postdysenteric arthritis) and nonspecific arthritis/arthralgia (N.A.A) (which included several related arthropathy and arthralgia diagnoses). Incidence was estimated using events and the total number of active duty personnel for each year.

**Results:**

506 cases of specific ReA were identified in active duty personnel between 1999 and 2007. Another 16,365 cases of N.A.A. were identified. Overall incidence was 4.1 (95% CI: 3.7, 4.5) and 132.0 (95% CI, 130.0-134.0) per 100,000 for specific ReA and N.A.A, respectively. Compared to the youngest age category, the incidence of both outcomes increased 7-fold with a concurrent increase in symptom duration for cases over the age of 40. Specific IGE exposures were documented in 1.4% of subjects. After adjusting for potential confounders, there was a significant association between IGE and ReA (specific reactive arthritis OR: 4.42, 95% CI: 2.24, 8.73; N.A.A OR: 1.76, 95% CI: 1.49, 2.07).

**Conclusions:**

Reactive arthritis may be more common in military populations than previously described. The burden of ReA and strong association with antecedent IGE warrants continued IGE prevention efforts.

## Background

Although infectious gastroenteritis (IGE) is typically a short-lived, acute illness, disease morbidity is significant. Incidence rates in travelers have been reported at 29 episodes per 100 person-months, based on self-reported symptoms and cohort studies[1]. Total IGE morbidity is likely underestimated due to unmeasured chronic sequelae, including reactive arthritis (ReA)[2-8]. ReA is a systemic inflammatory disorder characterized by an asymmetric, additive, aseptic arthritis triggered by an infection at a distant site. Previously considered a self-limited disease, the potential of ReA to cause prolonged, recurrent, or erosive disease has increasingly been recognized[2,3,5,7,9-11].

IGE-associated ReA is most commonly described after infection with *Campylobacter *[11-15], *Salmonella*[16-19], *Shigella*[4,20], and *Yersinia*[21,22]. Other ReA case reports have implicated asymptomatic enteric infections[23], enterotoxigenic *E. coli *(ETEC)[10,24], *Cryptosporidium *spp[25,26], *Giardia lamblia*[27], *Strongyloides stercoralis*[28], and possibly *Schistosoma mansoni*[23], and *Clostridium difficile*[29-33].

Two pseudonyms for ReA exist. Reiter's disease (RD) refers to the classic triad of arthritis, urethritis, and conjunctivitis, while postdysenteric arthritis (PA) refers specifically to IGE-related disease. In recent years, both subsets of disease have been referred to as reactive arthritis, although the naming convention has not been standardized[2,34]. Current ICD-9 coding includes PA and RD, but not ReA.

Prior studies based on culture-confirmed bacterial disease have estimated incidence rates of 3.1-5.0 per 100,000[35,36]. However, these estimates do not account for non-bacterial etiologies or uncultured IGE-associated ReA. A Scandinavian study showed a higher incidence (18 per 100,000) based on serologic and/or culture-based evidence of a prior infection[37].

The US military is at high risk of exposure to IGE-causing pathogens during deployment [1,38,39] and may subsequently be at an increased risk of ReA. The considerable health-care associated costs and decreased quality of life associated with ReA and other IGE sequelae could represent an important consideration to further justify efforts aimed at decreasing the incidence of IGE among US military.

## Methods

This was a matched case-control study to evaluate ReA associated with IGE in a US military population. Data were obtained from the Armed Forces Health Surveillance Center (AFHSC) which operates the Defense Medical Surveillance System (DMSS), a database containing medical data on all active duty US military personnel[40]. All records meeting outcome case definitions from 1999 to 2007 were included. These data were linked via social security number to demographic data from the Defense Manpower Data Center (DMDC), and deployment data. Data were compiled into a single dataset, de-identified, and provided to the investigators by AFHSC personnel. The number of active military personnel, stratified by service, year, and age, was obtained through the Defense Medical Epidemiology Database (DMED)[41]. The study protocol was approved by the Naval Medical Research Center and the Uniformed Services University of the Health Sciences Institutional Review Boards in compliance with all applicable Federal regulations governing the protection of human subjects.

### Outcome

The primary outcome of interest was ReA. Cases were identified using ICD-9 codes associated with ReA that met case definitions as described in Table 1. Since there is no ICD-9 code for 'reactive arthritis', and because RD has historically required the presence of cervicitis or urethritis, several nonspecific arthropathy and arthralgia related ICD-9 codes were included. Postdysenteric arthritis and RD cases were combined into a 'specific reactive arthritis' outcome. Cases meeting the nonspecific definition were termed 'nonspecific arthropathy/arthralgia' (N.A.A). To minimize incorrect or spurious diagnoses, our case definitions required a minimum of two separate ReA-related medical encounters within a 12 month period.

**Table 1:** Case definitions for primary outcomes

Category	ICD-9 code(s)	Case Definition
Reiter's Disease	099.3, 711.1	Minimum of two medical encounters with Reiter's disease coded within 12 months
		-OR-
		One medical encounter with Reiter's disease coded, preceded by at least one encounter with a nonspecific arthropathy coded within 12 months
Postdysenteric arthritis	711.3	Minimum of two medical encounters with postdysenteric arthritis coded within 12 months
		-OR-
		One medical encounter with postdysenteric arthritis coded, preceded by at least one encounter with a nonspecific arthropathy coded within 12 months
Specific ReA diagnosis	099.3, 711.1, 711.3	Meets criteria for Reiter's Disease OR postdysenteric arthritis
Nonspecific arthropathy/arthalgia^a^	716.4, 716.5, 716.6, 716.9, 719.4	Minimum of two medical encounters with a nonspecific arthropathy coded within 12 months. Subjects meeting the Reiter's disease or postdysenteric arthritis definitions were excluded.

a The listed nonspecific arthopathy/arthalgia ICD-9 codes correspond to the following: transient arthropathy (all subgroups), unspecified polyarthropathy or polyarthritis (all subgroups), unspecified monoarthritis (all subgroups), other specified arthropathy (all subgroups), and unspecified arthropathy (all subgroups)

ReA cases with a concurrent diagnosis of chlamydia or gonococcal disease (within 6 months; by ICD-9 code) were excluded. All other ReA cases were included in the analysis. Each case was matched by year of birth, sex, and calendar year to 4 controls with unrelated medical encounters.

The number and duration of ReA-related clinical visits were determined using the number of medical encounters with a ReA-related ICD-9 and time between the first and last ReA-related visit, respectively. When available, the affected site was also recorded (Reiter's disease ICD-9 code 099.3 does not include a sub-code to indicate affected site).

### Exposure

The primary exposure of interest was infectious gastroenteritis (IGE). Prior surveys have indicated that stool cultures are requested in less than 50% of outpatient visits for acute diarrhea[42]. As such, exposures were evaluated both for specific pathogens, and for nonspecific infectious enteritis as previously described[43]. Exposures were determined by ICD-9 codes for clinical visits within a 6-month window prior to case-presentation or censoring.

### Potential confounders

Demographic variables such as race, rank, marital status, educational level, and branch of service were evaluated as potential confounders. Deployment to an area of high traveler's diarrhea risk within 6 months of censure was also evaluated as a surrogate for IGE. High-risk deployments were defined as Operation Iraqi Freedom, Operation Enduring Freedom, the Persian Gulf, and/or Southwest Asia.

### Analysis

Crude incidence rates were calculated using the number of incident ReA cases, and active duty population figures. Confidence intervals for rates were computed using exact binomial methods. Analysis of change in incidence rates over time was completed using a Poisson regression. A Pearson's Chi-square test was used to compare the proportion of cases receiving care at given time-points after initial diagnosis. The association between ReA, IGE, and potential covariates was assessed using univariate and multivariate conditional logistic regression models. For the multivariate models, a backwards elimination approach was used and variables were dichotomized based on univariate analyses. The variable with the largest p-value was removed, and the model was then re-fit. This process was continued iteratively until all variables retained in the models were significant at an alpha = 0.15. Separate analyses were performed for the specific ReA diagnosis (RD and PA) and the nonspecific diagnosis. Two-tailed statistical significance was evaluated using an alpha of 0.05. Statistical analyses were performed using SAS v. 8.2 for Windows (SAS Institute, Cary, NC).

## Results

**Figure 1:**
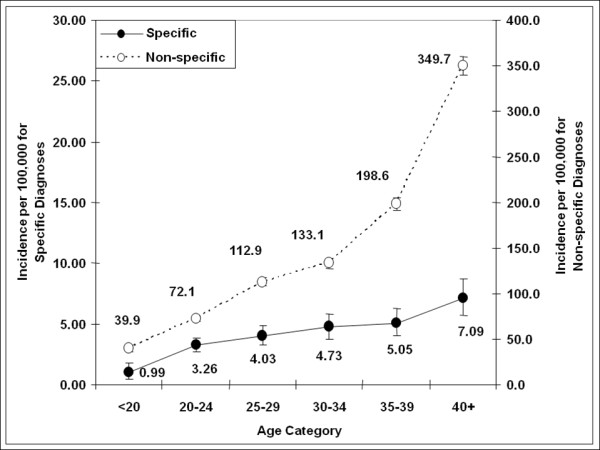
**ReA incidence rate (95%CI) by age, category, and diagnosis**.

Between 1999 and 2007 a total of 506 subjects met the specific ReA case definition, including 482 with Reiter's disease (RD) and 32 with postdysenteric arthritis (PA). Eight cases met both the RD and PA case definitions. An additional 16,365 cases met the nonspecific arthritis/arthalgia (N.A.A) case definition. Incidence rates for specific ReA and N.A.A were 4.1 (95% CI: 3.7, 4.5) and 132.0 (95% CI: 130.0, 134.0) per 100,000, respectively. A rising incidence over time of N.A.A was observed (p < 0.0001), while incidence rates for specific ReA remained stable (Figure 1).

Demographic data for the included study population are shown in Tables 2 and 3. For RD and PA, cases were more commonly deployed to a high risk region in the 6 months pre-censure (7.5% and 9.4%, respectively) than controls (3.8% and 3.1%, respectively) (p = 0.001 and p = 0.14, respectively). The opposite was true for N.A.A, where controls were more likely to have been deployed (4.7%) than cases (2.3%) (p < 0.001). Only 1.4% of all cases had an exposure documented within the preceding 6 months, and very few of these indicated pathogen-specific causes. A greater proportion of RD and PA cases had a documented exposure (3.5% and 3.1%, respectively), compared to nonspecific ReA (1.3%) (p < 0.001 and p = 0.4, respectively). However, prior IGE was more common in all cases than in their matched controls.

**Table 2:** Demographic characteristics of US military service members diagnosed with Reiter's disease and postdysenteric arthritis between 1999 and 2007 and their matched controls

Category	Subcategory	Reiter's Disease Cases^a^	Reiter's Disease Controls	Postdysenteric Arthritis Cases^a^	Postdysenteric Arthritis Controls
**N**		**482**	**1,928**	**32**	**128**
**Age **(mean ± SD)		30.9 ± 8.1	30.9 ± 8.1	32.1 ± 8.61	32.1 ± 8.61
**Sex **(n; %)	Male	436 (90.5)	1,744 (90.5)	24 (75.0)	96 (75.0)
	Female	46 (9.5)	184 (9.5)	8 (25.0)	32 (25.0)
**Race **(n; %)	Asian	14 (2.9)	106 (5.5)	1 (3.1)	7 (5.4)
	Black	57 (11.8)	340 (17.6)	5 (15.6)	26 (20.3)
	Hispanic	34 (7.1)	187 (9.7)	2 (6.3)	16 (12.5)
	Indian	6 (1.2)	16 (0.8)	0 (0.0)	1 (0.8)
	White	356 (73.9)	1,235 (64.1)	23 (71.9)	71 (55.5)
	Other/Unknown	15 (3.1)	44 (2.3)	1 (3.1)	7 (5.5)
**Marital Status **(n; %)	Single	171 (35.5)	713 (37.0)	10 (31.3)	55 (43.0)
	Married	280 (58.1)	1,141 (59.1)	20 (62.5)	68 (43.0)
	Other/Unknown	31 (6.4)	74 (3.8)	2 (6.3)	5 (3.4)
**Service Branch **(n; %)	Air Force	142 (29.5)	459 (23.8)	14 (43.8)	37 (28.9)
	Army	156 (32.4)	729 (37.8)	11 (34.4)	54 (42.2)
	Marine	59 (12.2)	241 (12.5)	3 (9.4)	14 (10.9)
	Navy	125 (25.9)	499 (25.9)	4 (12.5)	23 (18.0)
**Rank **(n; %)	Enlisted	389 (80.7)	1,554 (80.6)	24 (75.0)	94 (73.4)
	Warrant	9 (1.9)	21 (1.1)	0 (0.0)	3 (2.3)
	Officer	84 (17.4)	353 (18.3)	8 (25.0)	31 (24.2)
**Education level **(n; %)	High School or equivalent	310 (63.9)	1,240 (64.3)	15 (46.8)	77 (60.2)
	Some college	63 (13.1)	218 (11.3)	7 (21.9)	11 (8.6)
	Bachelors	57 (11.8)	265 (13.7)	7 (21.9)	20 (15.6)
	Post grad	33 (6.8)	152 (7.9)	3 (9.4)	14 (10.9)
	Unknown	19 (3.9)	53 (2.7)	0 (0.0)	6 (4.7)
**High Risk Deployment^b^**	6 months	36 (7.5)	74 (3.8)	3 (9.4)	4 (3.1)
(n; %)	5 months	31 (6.4)	59 (3.1)	1 (3.1)	4 (3.1)
	4 months	22 (4.6)	49 (2.5)	1 (3.1)	2 (1.6)
	3 months	15 (3.1)	37 (1.9)	1 (3.1)	1 (1.6)
**Documented Infectious**	6 months	17 (3.5)	15 (0.8)	1 (3.1)	2 (1.6)
**Gastroenteritis **(n; %)	5 months	15 (3.1)	11 (0.6)	1 (3.1)	2 (1.6)
	4 months	14 (2.9)	10 (0.5)	1 (3.1)	0 (0.0)
	3 months	13 (2.7)	9 (0.5)	1 (3.1)	0 (0.0)

Backround active duty population rates: 85% male; 69%white/19%black/12%other; 35%Army/26%AirForce/26%Navy/13%Marine

^a ^Eight cases overlap case definitions for both Reiter's disease and postdysenteric arthritis

^b ^Deployment to a region at high risk for travelers' diarrhea (Iraq, Afghanistan, Persian Gulf or Southwest Asia) within the indicated number of months prior to ReA diagnosis (cases) or censoring (controls)

**Table 3:** Demographic characteristics of US military service members diagnosed with nonspecific arthropathy/arthralgia between 1999 and 2007 and their matched controls

Category	Subcategory	Nonspecific Arthralgia/ Arthropathy Cases	Nonspecific Arthralgia/ Arthropathy Controls
**N**		**16,365**	**65,422**
**Age **(mean ± SD)		33.4 ± 8.8	33.4 ± 8.8
**Sex **^a ^(n; %)	Male	12,979 (79.3)	51,895 (79.3)
	Female	3,385 (20.7)	13,523 (20.7)
**Race **(n; %)	Asian	620 (3.8)	2914 (4.5)
	Black	3,687 (22.5)	12,256 (18.7)
	Hispanic	1,445 (8.8)	6,290 (9.6)
	Indian	250 (1.5)	922 (1.4)
	White	9,920 (60.6)	41,210 (63.0)
	Other/Unknown	443 (2.7)	1,830 (2.8)
**Marital Status **(n; %)	Single	4,407 (26.9)	20,536 (31.4)
	Married	10,904 (66.6)	41,398 (63.3)
	Other/Unknown	1,054 (6.4)	3488 (5.3)
**Service Branch **(n; %)	Air Force	3,671 (22.4)	17,089 (26.1)
	Army	7,865 (48.1)	24,628 (37.6)
	Marine	1,016 (6.2)	6,350 (9.7)
	Navy	3,813 (23.3)	17,355 (26.5)
**Rank ^a^**(n; %)	Enlisted	13,782 (84.2)	50,541 (77.2)
	Warrant	312 (1.9)	1,106 (1.7)
	Officer	2,271 (13.9)	13,775 (21.1)
**Education level **(n; %)	High School or equivalent	10,781 (65.9)	39,195 (59.9)
	Some college	2,023 (12.4)	7,563 (11.6)
	Bachelors	1,835 (11.2)	9,511 (14.5)
	Post grad	1,330 (8.1)	7,204 (11.0)
	Unknown	396 (2.4)	1,949 (3.0)
**High Risk Deployment^b^**	6 months	374 (2.3)	3,097 (4.7)
(n; %)	5 months	280 (1.7)	2,571 (3.9)
	4 months	182 (1.1)	2,077 (3.2)
	3 months	110 (0.7)	1,568 (2.4)
**Documented Infectious**	6 months	211 (1.3)	472 (0.7)
**Gastroenteritis **(n; %)	5 months	181 (1.1)	401 (0.6)
	4 months	154 (0.9)	332 (0.5)
	3 months	123 (0.8)	256 (0.4)

Backround active duty population rates: 85% male; 69%white/19%black/12%other; 35%Army/26%AirForce/26%Navy/13%Marine

^a ^Missing values: Sex (1 case, 4 controls), Rank (1 control)

^b ^Deployment to a region at high risk for travelers' diarrhea (Iraq, Afghanistan, Persian Gulf or Southwest Asia) within the indicated number of months prior to ReA diagnosis (cases) or censoring (controls)

**Figure 2:**
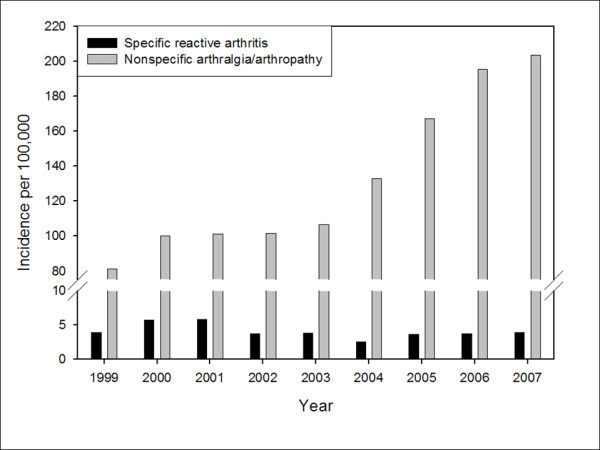
**Incidence of reactive arthritis and nonspecific arthropathy/arthralgia in active US military personnel 1999 to 2007**.

For both specific ReA and N.A.A, incidence rose with increasing age (Beta = 1.04, p = 0.002 and Beta = 55.68, p = 0.006, respectively). Distribution of cases by age category is shown in Figure 2. Nonspecific arthritis/arthralgia rates increased nearly 10-fold over the age spectrum, from 39.8 per 100,000 (95%CI 36.1-44.0) in the < 20 age group, to 349.7 per 100,000 (95%CI 339.5-360.2) in the > 40 age group. Similar increases were seen for specific ReA diagnoses, from 0.99 per 100,000 (95%CI 0.52-1.82) in the < 20 age group, to 7.09 per 100,000 (95% CI 5.70 - 8.72) in the > 40 age group.

Unadjusted and adjusted odds ratios (OR) with 95% confidence intervals (95% CI) are shown in Table 4. After controlling for deployment, branch of service, marital status, and rank, ReA cases had higher odds of documented IGE exposure compared to matched controls for both specific ReA (OR: 4.42; 95% CI: 2.24, 8.73) and N.A.A (OR: 1.76; 95% CI: 1.49, 2.07).

**Table 4:** Unadjusted and adjusted^a ^odds ratios (95% confidence intervals) for exposure variables and included covariates from a conditional logistic regression model evaluating the risk of specific reactive arthritis and non-specific arthralgia/arthropathy among active duty US military personnel from 1999 to 2007

Model	Variable	All Specific ReA	Nonspecific Arthralgia/Arthropathy
**Univariate**	**Prior exposure**^b^	4.50 (2.30, 8.82)	1.80 (1.53, 2.12)
	**High risk deployment**^c^	2.12 (1.40, 3.21)	0.47 (0.42, 0.52)
	**Army service**	0.77 (0.63, 0.95)	1.54 (1.48, 1.59)
	**Married**	0.96 (0.76, 1.22)	1.22 (1.17, 1.27)
	**Non-white**	0.63 (0.50, 0.78)	1.11 (1.07, 1.15)
	**Enlisted rank**	1.04 (0.79, 1.37)	1.68 (1.60, 1.76)
**Multivariate**	**Prior exposure**^b^	4.42 (2.24, 8.73)	1.76 (1.49, 2.07)
	**High risk deployment**^c^	2.07 (1.36, 3.15)	0.46 (0.41, 0.51)
	**Army service**	0.77 (0.62, 0.94)	1.54 (1.49, 1.60)
	**Married**	0.97 (0.76, 1.23)	1.21 (1.16, 1.27)
	**Enlisted rank**	1.03 (0.78, 1.36)	1.66 (1.58, 1.74)

^a ^Adjusted for all covariates retained in the final model (prior exposure, high risk deployment, Army service, married, non-white and enlisted rank)

^b ^Defined as an infectious diarrhea diagnosis six months prior to ReA diagnosis (cases) or censoring (controls)

^c ^Defined as deployment to a region at high risk for travelers' diarrhea (Iraq, Afghanistan, Persian Gulf or Southwest Asia) six months prior to ReA diagnosis (cases) or censoring (controls)

Deployment to a high risk area was associated with an increased risk of specific ReA (OR: 2.07; 95% CI: 1.36, 3.15). However, it was protective against N.A.A (OR: 0.47; 95% CI: 0.41, 0.51). Army service (compared to other branches of service) was associated with an increased risk of N.A.A (OR 1.54; 95%CI: 1.49, 1.60). This association was reversed for specific ReA (OR: 0.77; 95% CI: 0.62, 0.94). Odds ratios for marriage and enlisted status in the N.A.A group were 1.21 (95% CI: 1.16, 1.27) and 1.66 (95% CI: 1.58, 1.74), respectively. Neither marital status nor rank were associated with a change in specific ReA risk.

**Figure 3:**
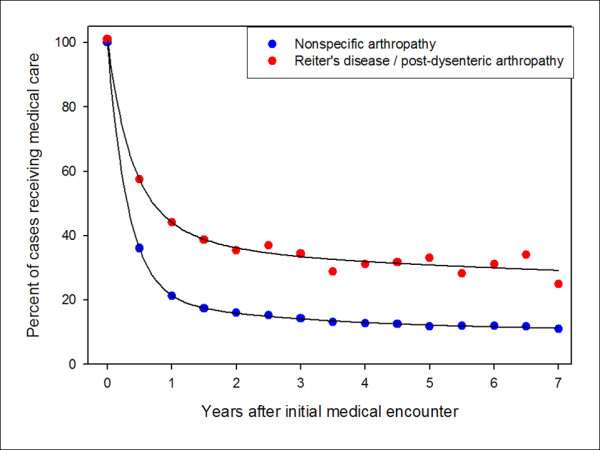
**Duration of reactive arthritis-associated medical care in active US military personnel 1999 to 2007**.

Duration of ReA symptoms was prolonged after initial diagnosis. Among specific ReA cases that remained on active duty, 35.5% (89/251) were still receiving ReA-related medical care for a minimum of 2 years after initial presentation (Figure 3). This proportion was lower (p < 0.001) for the N.A.A cases (1,019/6,380; 16.0%). Increased duration of care was associated with increasing age for both the specific ReA (Cochran Armitage Trend p < 0.001) and non-specific (p < 0.001) arthropathy/arthralgia outcomes with the following proportion receiving care at least 2 years after initial diagnosis (specific ReA: ≤ 25: 13.5%, > 25 to < 35: 29.2%, ≥35: 57.3%; N.A.A: ≤ 25: 12.7%, > 25 to < 35: 28.5%, ≥35: 58.9%). Males had significantly higher proportion receiving care for specific ReA at 2 years than did females (38.7% vs 7.7%, respectively) (Pearson Chi-Square p = 0.002). This gender effect was not observed for the N.A.A outcome.

An analysis of the affected site showed that the majority (47%) of cases had no indicated joint. Although categories are not mutually exclusive, RD (limited to ICD-9 code 711.1) predominately affected the knee (9.8%) or multiple joints (17.1%). The hand (15.6%), knee (15.6%), and multiple joints (28.1%) were the most commonly affected in PA. N.A.A most commonly affected the shoulder (22.6%), knee (15.3%), and ankle/foot (13.0%), with only 2.4% coded as multiple joints.

## Discussion

We found an incidence of 4.1 per 100,000 persons for specific ReA in an active duty population. Prior publications have reported similar population incidence rates of 3-5 per 100,000, for ReA with culture confirmed antecedent bacterial IGE[35,36]. However, as clinical recognition of antecedent infection is required for the specific diagnoses, both the incident rate we report and prior published rates for specific ReA likely underestimate the true incidence of IGE-associated ReA.

Prior studies have reported undifferentiated arthritis in an attempt to describe cases that do not meet the full diagnostic criteria for RD or PA. Schillerup *et al*. reported an incidence of 41 per 100,000 for undifferentiated inflammatory arthritis[44], and Fendler *et al*. described a ReA case series in which 59% of patients presented with a undifferentiated oligoarthritis[45]. Similarly, we used a N.A.A case definition and found an incidence of 132 per 100,000. Our N.A.A. case definition is overly sensitive due to limitations associated with the use of ICD-9 codes. It is very possible that a proportion of our N.A.A cases are somehow different than the specific ReA cases. However, our description of these cases illustrate an upper bound to the incidence of disease to be used in conjunction with the lower bound obtained from the highly restrictive postinfectious gastroenteritis and Reiter's disease case definitions. We suspect the large difference in incidence rates indicate there is a lack of clinical recognition of the reactive nature of at least some arthritis/arthropathy cases. Of note, we found an increasing incidence of N.A.A cases from 2002 to 2007 with an unknown etiology. The impact of deployment tempo during this time frame on N.A.A incidence could not be determined, as only data on deployment to certain high-risk areas was obtained.

The incidence of both specific ReA and N.A.A increased with age. Published descriptions of age in ReA indicate most cases occur in a young population, generally less than 40 years of age[2,23]. This description likely stems from the earliest case reports of ReA, which largely consisted of STD-related cases. We found no prior publications reporting an increasing incidence with age. Further evaluation of the incidence of ReA in an older population would be worthwhile; however our use of an active duty study population limits our ability to do so.

As expected, we found increased odds of prior IGE in ReA cases compared to matched controls, despite the low rate of documented antecedent gastroenteritis. After controlling for covariates, the OR for specific ReA was 4.42 (95% CI: 2.24, 8.73). The effect was smaller in the N.A.A group (OR: 1.76, 95% CI: 1.49, 2.07), though it remained statistically significant. Our actual number of documented cases of IGE was very low; however relying on medical records for documentation of IGE severely underestimates the true incidence[42]. A prior population-based survey indicated less than a quarter of those with acute diarrheal illness seek medical care[46]. This is clearly evident in the paucity of documented IGE exposures in our cases diagnosed with RD and PA, both of which require a clinical recognition of antecedent GI disease.

Our cases are not strictly limited to IGE-related ReA. While we excluded ICD-9 documented Chlamydia and gonococcal infections, our methods potentially misses both asymptomatic chlamydial infections and STI cases diagnosed outside of military data catchment (such as through a public health department). However, IGE related disease likely comprises a large proportion of all reactive arthritis cases, as has been reported previously[37].

We assessed the effect of deployment to high traveler's diarrhea risk regions as a potential surrogate for IGE, due to incomplete data capture in a deployed population. We found an increased odds of prior deployment in those with specific ReA diagnoses. Conversely, there was a protective effect of deployment for the N.A.A diagnoses. The etiology of this relative difference is unclear, but the unique difficulties in capture of deployment related illness and injury data may account for the qualitative shift in the effect estimate. More severe injuries may be preferentially captured as those patients reach higher levels of care where integrated electronic medical systems are more prevalent. Additionally, stress injuries such as tendonitis, trauma, etc are commonly expected when deployed. Patients may be given one of these more common diagnoses, rather than one of the nonspecific codes used in our N.A.A. case definition. Finally, military members with underlying medical illness may not be deployed resulting in a type of "healthy worker" effect which might also explain the lower rate of diagnoses in deployed personnel.

Inherent difference between the specific ReA and N.A.A group could account for directional differences in some effect estimates noted above. This is supported by the difference in concurrent diagnoses for the two case definitions. Specifically, approximately 13% of the N.A.A cases were seen for physical therapy, 4% for lumbago, and 3% for aftercare following surgery. None of these co-morbid diagnoses were among the top 20 for the specific ReA cases. Interestingly, functional gastrointestinal diagnoses were prevalent in both groups (data not shown).

Although the original reports of ReA indicated a self-limited disease, recent publications have described chronic disease in up to half of cases, especially in the presence of HLA-B27[9,34,47]. Our data supports the idea of a more chronic disease, especially among patients over the age of 35. While we were unable to assess the symptoms associated with a ReA-related medical encounter, or HLA-B27 status, ReA continued to be commonly coded as a primary diagnosis up to 7 years after the initial medical encounter. The chronicity of this disease has not been well recognized by clinicians. However, like the functional gastrointestinal postinfectious disorders, the morbidity associated with ReA needs to be considered by providers and policy makers when assessing the impact associated with deployment of countermeasures, in order to mitigate the effects of acute enteric infections during deployment.

The U.S. active duty military population was used as the study population. There are several unique qualities of this population that should be considered when comparing our findings to the general population. The military population is younger (the average age is roughly 28) and generally physically fit, yet extensive physical stressors likely increase the number of musculoskeletal injuries in this population. Military members may travel extensively to underdeveloped areas of the world, placing them at increased risk for IGE exposures. In addition, as there is a direct link between health and duty status, deployment eligibility, and disability claim eligibility, members may have additional ICD-9 coded visits to address the administrative aspects of health issues.

Utilization of a medical encounter database for epidemiologic studies carries an intrinsic risk of misclassification or systemic biases, in addition to those described specifically above. Multiple studies have demonstrated inaccuracies in ICD-9 coding[48-50]. As described in previous publications, we required multiple medical encounters for the same diagnosis in an attempt to minimize case misclassification[43,51].

## Conclusion

In summary, we found that ReA was more prevalent than previously recognized in the U.S. active duty military population, especially when non-specific ReA cases were considered. This represents a significant burden on the military healthcare system and may be an important medical condition in returning veterans. Due to the strong association between IGE and ReA as well as other post-infectious sequelae, and the high risk of travelers diarrhea among deployed US service members, efforts aimed at minimizing IGE incidence in DoD personnel are warranted and support enhancement of current primary prevention strategies and development of novel solutions.

## Competing interests

The authors declare that they have no competing interests.

Authors are employees of the U.S. Government and military service members. This work was prepared as part of official duties. Title 17 U.S.C. §105 provides that 'Copyright protection under this title is not available for any work of the United States Government.' Title 17 U.S.C. §101 defines a U.S. Government work as a work prepared by a military service member or employee of the U.S. Government as part of that person's official duties. The opinions and assertions herein should not be construed as official or representing the views of the Department of the Navy, Department of the Army, the Department of Defense, or the US Government. This is a US Government work. There are no restrictions on its use.

## Authors' contributions

JC assisted with the statistical analysis and drafted the manuscript. RG conceived of the study and participated in its design. MR participated in the study design, assisted in coordination of the study, and helped draft the manuscript. DT participated in the study design and helped draft the manuscript. CP participated in the study design, assisted in coordination of the study, performed the statistical analysis, and helped draft the manuscript. All authors read and approved the final manuscript.

## Pre-publication history

The pre-publication history for this paper can be accessed here:

http://www.biomedcentral.com/1471-2334/10/266/prepub
